# Cost-Effective Multitask Active Learning in Wearable Sensor Systems

**DOI:** 10.3390/s25051522

**Published:** 2025-02-28

**Authors:** Asiful Arefeen, Hassan Ghasemzadeh

**Affiliations:** 1 College of Health Solutions, Arizona State University, Phoenix, AZ 85004, USA; 2 School of Computing and Augmented Intelligence, Arizona State University, Tempe, AZ 85281, USA

**Keywords:** active learning, activity recognition, digital health, mobile health, multi-task learning, stress monitoring

## Abstract

Multitask learning models provide benefits by reducing model complexity and improving accuracy by concurrently learning multiple tasks with shared representations. Leveraging inductive knowledge transfer, these models mitigate the risk of overfitting on any specific task, leading to enhanced overall performance. However, supervised multitask learning models, like many neural networks, require substantial amounts of labeled data. Given the cost associated with data labeling, there is a need for an efficient label acquisition mechanism, known as multitask active learning (MTAL). In wearable sensor systems, success of MTAL largely hinges on its query strategies because active learning in such settings involves interaction with end-users (e.g., patients) for annotation. However, these strategies have not been studied in mobile health settings and wearable systems to date. While strategies like one-sided sampling, alternating sampling, and rank-combination-based sampling have been proposed in the past, their applicability in mobile sensor settings—a domain constrained by label deficit—remains largely unexplored. This study investigates the MTAL querying approaches and addresses crucial questions related to the choice of sampling methods and the effectiveness of multitask learning in mobile health applications. Utilizing two datasets on activity recognition and emotion classification, our findings reveal that rank-based sampling outperforms other techniques, particularly in tasks with high correlation. However, sole reliance on informativeness for sample selection may introduce biases into models. To address this issue, we also propose a *Clustered Stratified Sampling* (CSS) method in tandem with the multitask active learning query process. CSS identifies clustered mini-batches of samples, optimizing budget utilization and maximizing performance. When employed alongside rank-based query selection, our proposed CSS algorithm demonstrates up to 9% improvement in accuracy over traditional querying approaches for a 2000-query budget.

## 1. Introduction

Traditional supervised learning models, designed for solving single tasks like forecasting, regression, and classification on sensor data, have shown effectiveness for decades [1,2,3]. However, acquiring labels for sensor data largely depends on input from end users. Furthermore, efficiency of these supervised learning methods declines when confronted with multiple tasks simultaneously, as these models overlook cross-task shared information that can be leveraged for improved performance [4,5]. Recognizing this limitation, recent research has explored advanced models aiming for enhanced performance, efficiency, and adaptability [6,7,8,9,10]. Therefore, multitask learning models (MTLs) have emerged as a viable alternative, directly challenging the conventional approach of employing distinct models for each task  [11].

Leveraging the shared representation of tasks, MTLs learn to mitigate the risk of overfitting up to a certain sharing limit. Adding more tasks into these models reduces the likelihood of overfitting [5]. Nevertheless, dealing with a set of unrelated tasks can result in negative knowledge transfer, leading to a subpar performance [12]. In contrast, for a set of related tasks, MTL offers several advantages, including (i) enhanced accuracy through inductive knowledge transfer and the utilization of auxiliary information, (ii) a more compact architecture, (iii) reduced computational costs, and (iv) swift operation—especially beneficial for tasks requiring low latency, such as mobile health applications [12,13]. These advantages place MTL as a superior alternative to deploying multiple single-task models.

MTLs, like other supervised machine learning models, require a significant amount of labeled data. While the deployment of a multi-task learning model is highly sought after, a notable challenge lies in providing the model with the necessary labeled data. However, acquiring such labeled data can be challenging due to the associated expense, tedium, and time-consuming nature [14]. To address this challenge, one approach involves integrating the MTL into an active learning loop, where it receives a strategically chosen subset of labeled data. In this setup, the MTL collaborates with an active learning agent within the multitask active learning (MTAL) ecosystem, aiming for high performance with substantially less data. The agent identifies informative samples located near the decision boundary, where the model is least confident [15]. These critical data instances are presented to an oracle for labeling and the iterative process continues until the budget is depleted or a predefined accuracy threshold is attained.

In MTAL, we usually pick the most useful samples using strategies like *one-sided*, *alternating*, and *rank combination* (*RC*)-based selection, as well as criteria such as *least confidence*, *margin sampling*, and *entropy* [16,17,18,19]. These strategies are effective in situations where there are limited data available [20]. However, as far as we are concerned, they have not been tested in mobile health (mHealth) settings. This technology is relatively new and constrained with challenges, like having very little initial training data and a limited annotation budget [21].

**Figure 1:**
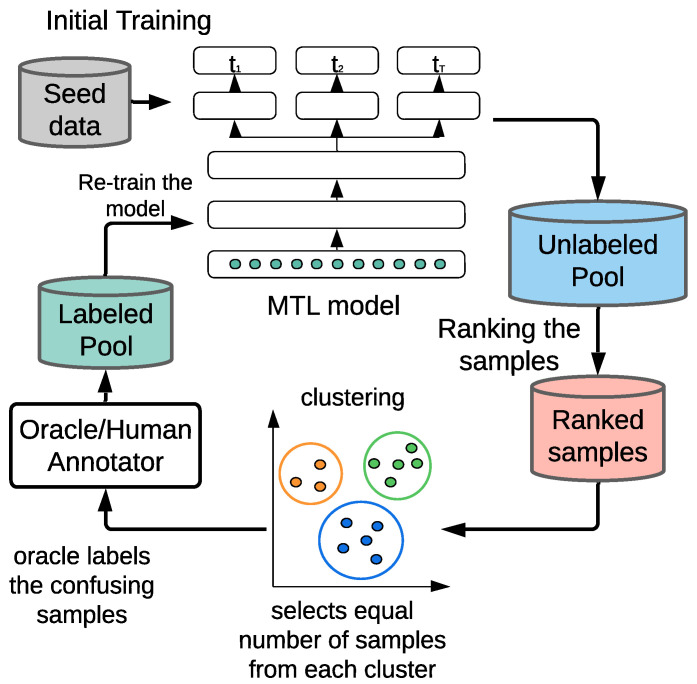
Proposed multitask active learning loop that ensures the model receives equal number of labeled instances from each cluster during re-training.

To counter the limitation of the current MTAL framework in wearable sensor systems such as in mHealth settings, we propose a clustered stratified sampling (CSS) algorithm (Figure 1) that works with these query schemes and ensures effective training of the MTLs. In CSS, our goal is to empower the predictive model, leveraging a clustering technique within the active learning loop such that the model performs better in a substantially low-seed-data environment with small querying budget. We show that although training with the CSS algorithm introduces additional computation, it ensures a substantial performance boost. Because training in mobile health can be performed between microinteraction-based Ecological Momentary Assessment (µEMA) [22] prompts, this allows us enough time to train the model.

Our contributions are summarized as follows:

- First, we study the utility of multitask active learning querying strategies including one-sided selection, alternating selection and RC-based selection under a mobile health setup. To this end, we construct two datasets for multitask learning in mobile health settings and demonstrate the performance of the query strategies.
- Second, we introduce a *Clustered-Stratified Sampling* (*CSS*) algorithm and employ it in parallel with the query methods to boost the performance compared to their original forms. We show through analysis that CSS exhibits substantial performance elevation for multiple tasks related to human activity and emotion classification from wearable sensor-transmitted signals and brain signals, respectively.

## 2. Related Work

Query strategies are a fundamental part of active learning or multitask active learning since they help to choose the most critical data samples efficiently.

### 2.1. Unlabeled Data Volume

Pool-based querying [23], membership, disjoint, equivalent queries [24] and stream-based sampling [25,26] are introduced in prior research. Stream-based sampling decides if any incoming sample is to be sent to the oracle for labeling, whereas pool-based sampling iteratively discovers the best instances from an unlabeled pool using newly gained knowledge [27]. Recent work has focused on leveraging multitask learning frameworks for more effective pool selection by incorporating outlier detection and multi-scale scoring. For instance, Zhang et al. [28] introduced an outlier-aware multitask learning method to model inliers and detect anomalies in sound data. This approach highlights the potential of incorporating anomaly detection into multitask active learning to improve the robustness of data selection strategies. Similarly, advancements in multitask learning for speech translation tasks, such as those proposed by Zhang et al. [29], demonstrate the importance of task-specific adaptations for improving overall system performance. Uncertainty-guided techniques, such as probabilistic transformers, have been employed in domains like complex action recognition, where tasks often rely on shared features extracted from high-dimensional sequences [30]. Furthermore, Wang et al. [31] proposed an uncertainty sampling approach for action recognition that maximizes the expected average precision to showcase the effectiveness of uncertainty-based strategies in identifying the most valuable data points for labeling. These methods reinforce the role of task interdependence and tailored modeling techniques in improving the data selection and learning in multitask systems.

### 2.2. Informativeness Estimation

In informativeness estimation, the goal is to prioritize data samples that provide the most value for learning, particularly in multitask scenarios where tasks share information but also introduce unique challenges. Earlier works [32,33] exploited Support Vector Machine (SVM) and probability-based estimation to discover informative samples. SVM-based active learning [34] relies on margin sampling to identify uncertain data points, while Tong et al. [35] proposed iterative methods that adapt as new knowledge is incorporated. These methods were primarily designed for single-task and often binary classification scenarios, which limit their applicability to multitask settings [36,37,38].

Recent works in multi-task active learning have introduced new strategies to extend these principles to multitask problems. For instance, Ref. [39] proposed a multitask learning approach with a focus on improved confidence bounds for active learning in multitask settings. In a similar vein, Ref. [40] emphasized the importance of consistency in multi-task active learning and proposed methods that ensure task coherence during sample selection. Ref. [40] introduced the concept of task consistency–diversity and was able to enhance the performance of multi-task networks during active learning. Furthermore, Ref. [41] explored the use of inconsistency-based multi-task cooperative learning in the context of emotion recognition, which highlights the benefits of cooperative learning strategies in multitask active learning.

### 2.3. Algorithm-Based Methods

Reichart et al. proposed *one-sided selection*, *alternating selection* and *rank combination* (*RC*) query strategies. The underlying theme is estimating informativeness based on a reference task (one-sided) or rotating the reference task (alternating) or sorting the samples based on their cumulative criticalness (RC). Ref. [42] selects representative and discriminative samples alternately using semi-supervised training to avoid training the external classifier multiple times on the same data. Ref. [43] proposed a novel way to estimate the informativeness of each multi-label sample from the unlabeled pool and utilized label hierarchies of samples for cost-effective querying. A clustering ensemble framework combining local and global uncertainties was proposed in [44] to identify uncertain data points for developing distinct clusters. Some of these methods used sample informativeness and were successful in the MTAL framework. Later, Ref. [45] focused on MTAL-based deep image classification and proposed a novel cost-effective active learning (CEAL) procedure. Unlike other techniques, CEAL allows the deep learning model to make predictions on the unlabeled pool, selects the most confident samples based on that, and assigns pseudo-labels. This means that the model is assumed to be confident enough to label unseen data. However, neural networks are greedy for labeled data, and there is a performance-related risk associated with the selection process when the initial seed data are minimal and the querying budget is also limited, which are common traits in mobile health settings.

## 3. Approach

Let {$t_{1}$, ⋯, $t_{T}$} be a set of *T* learning tasks in a multi-task active learning setting. Furthermore, let $L^{\left(i\right)}$ = {($x_{1}$, $y_{1}^{\left(i\right)}$), ($x_{2}$, $y_{2}^{\left(i\right)}$), ⋯, ($x_{n}$, $y_{n}^{\left(i\right)}$)} be the training set of size *n* for task $t_{i}$. Note that the input observations $x_{j}$ are shared across all the tasks. Each input observation $x_{j}$ is a *D*-dimensional vector of form $x_{j}$ = [$x_{j,1}$, $x_{j,2}$, ⋯, $x_{j,D}$], which yields $x\in R^{n\times D}$ as the feature set or raw sensor data and $y^{\left(i\right)}\in R^{n\times 1}$ as the target vector for task $t_{i}$. Similarly, the unlabeled pool $U^{m}$ contains *m* samples, where each sample is also a *D*-dimensional input. The target vector is an m×1 vector unknown to us.

Let ${\hat{y}}^{\left(i\right)}\in R^{p\times 1}$ be the prediction vector on test data for task $t_{i}$; we can write the loss for classification task $t_{i}$ using cross-entropy as follows.

(1) $$L\left({\hat{y}}^{\left(i\right)},y^{\left(i\right)}\right)=-\sum_{j=1}^{p}\sum_{c=1}^{C_{i}}y_{j}^{(i)c}\log\left(f\left({\hat{y}}_{j}^{(i)c}\right)\right)$$

where $C_{i}$ refers to the number of classes associated with task $t_{i}$, *p* represents the number of test samples, and f(·) denotes the output of the activation function.

The main objective of our multitask active learning is to minimize the total loss $L_{\left\{1,\cdots ,T\right\}}$ over the *T* tasks given an upper bound, *B*, on the number of queries that are made.

(2) $$Minimize\;\;\;\sum_{i=1}^{T}\alpha_{i}L\left({\hat{y}}^{\left(i\right)},y^{\left(i\right)}\right)$$

subject to:

(3) B≤λ

where $\alpha_{i}$ is the weight of task $t_{i}$ and λ is the maximum allowed queries that can be made. Solving the optimization problem in (2) will result in finding *W*, the parameters of the model.

Given the labeled pool L composed of sensor observations and task-specific labels, an active learning agent initializes training the model. With the obtained knowledge, the model is used to identify the most critical samples from the unlabeled pool as estimated by their informativeness. Informativeness can be estimated for each sample based on the least confidence [26] method:

(4) $$x_{LC}=\underset{x_{i}}{argmin}\;max\;p({\hat{y}}^{c}|x_{i})$$

or by using margin sampling [26]:

(5) $$x_{MS}=\underset{x_{i}}{argmin}\;\left[p\left({\hat{y}}^{1}|x_{i}\right)-p\left({\hat{y}}^{2}|x_{i}\right)\right]$$

or by Shannon’s information entropy [46]:

(6) $$x_{entropy}=\underset{x_{i}}{argmax}\;\left[-\sum p\left({\hat{y}}^{c}|x_{i}\right)\cdot \log\left(p\left({\hat{y}}^{c}|x_{i}\right)\right)\right]$$

**Figure 2:**
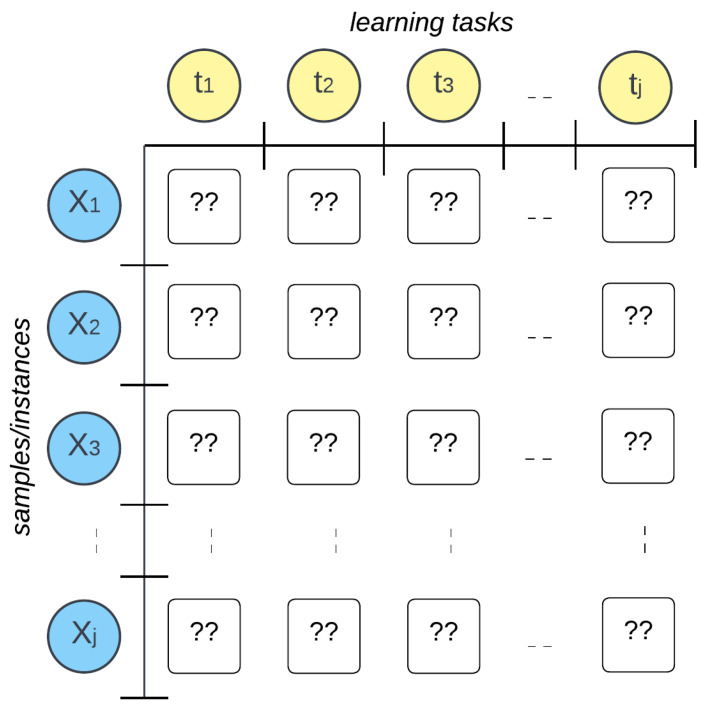
Choice board for querying in MTAL. Unlabeled samples can be labeled by setting a reference task, by altering the reference task periodically or by assigning a cumulative rank based on the uncertainty of each sample.

The critical samples, also known as the samples on which the model is least confident, are identified to be labeled by a human annotator or oracle. There are multiple choices regarding the direction along which the informativeness can be estimated. As referred to in Figure 2, informativeness can be estimated by considering a reference task and based on the prediction probabilities on $U^{m}$ made by the model for that specific task only. Alternatively, the reference task can be altered periodically rather than focusing on one task. Cumulative informativeness can be used as well to sort out the critical samples.

### Clustered Stratified Sampling (CSS)

After initial training with seed data (i.e., $|L^{n}|\approx 0$), a reference task is set in a typical one-sided selection and alternating selection method or not set at all for RC sampling. The model makes predictions on the unlabeled samples, and using their informativeness, the most critical data points are sorted out.

Setting the selection dependency solely based on informativeness, the agent will end up selecting samples abruptly distributed across different classes, thus biasing towards a specific class. Therefore, we aim to select samples evenly across the classes to alleviate the possibility of biasing towards any specific class. To this end, we employ a clustering method on the unlabeled pool to form clusters of similar data points. We then utilize the *least confidence* (*LC*) method to identify an equal number of the most critical data points by least top probabilities from these clustered samples. Note that CSS can be easily applied to the reference task-based approaches (i.e., one-sided and alternating) by simply setting the number of clusters equal to the number of classes of the reference task; however, there is no reference task in RC sampling, which leaves no clue for setting the number of clusters. Hence, we propose to relax the complexity of the approach and choose cluster number on the basis of the task with maximum classes.

This is also an interactive approach between the active learning agent and the oracle, where the agent repeatedly introduces these equally distributed highly informative samples to the oracle and gets them labeled. At each iteration, a mini-batch of *b* samples will be pulled from the unlabeled pool $U^{m}$ and integrated into the labeled pool $L^{n}$ after annotation. So, the $q^{th}$ iteration can be defined as:

(7) $$f_{q}:U^{m-qb}\overset{b\;samples}{\to }L^{n+qb}$$

Since we are developing clusters or strata of samples and performing stratified sampling, we call this method *Clustered Stratified Sampling* (*CSS*) [47].

Algorithm 1 and the following lemma are presented for better explaining the methods.
**Algorithm 1** Algorithm for *Clustered Stratified Sampling*-based sample selection**Inputs:**unlabeled pool, $U^{m}=\{x_{1},x_{2},\cdots$, $x_{m}\}$labeled pool, $L^{n}=$$\{\langle x_{1},\left(y_{1}^{\left(1\right)},y_{1}^{\left(2\right)},\cdots \right)\rangle ,\cdots$, $\langle x_{n},\left(y_{n}^{\left(1\right)},y_{n}^{\left(2\right)},\cdots \right)\rangle \}$Querying budget, *B*Querying batch size, *b***Ensure:** $|L^{n}|\approx 0$**Outputs:**multitask network trained with fewer datapointsTrain an multitask model $W^{0}$ from $L^{n}$$W^{i}\leftarrow W^{0}$k←0**while** k·b<B **do** Identify informative instances $X^{**}\in U$ using $W^{i}$ Arrange $X^{**}$ in $C_{\max}=\max_{i}C_{i}$ clusters Create $X^{*}$ by selecting an equal number of samples from each cluster Query the oracle to obtain label $Y^{*}$ of $X^{*}$ $L^{n+b}\leftarrow L^{n}\cup \langle X^{*},Y^{*}\rangle$ $U^{m-b}\leftarrow U^{m}\setminus \langle X^{*},Y^{*}\rangle$ Train multitask model $W^{i}$ with $L^{n+b}$ k←k+1**end while**Train final multitask network $W^{f}$ with $L^{n+k\cdot b}$

**Lemma** **1.**  *The time complexity of iterative Clustered Stratified Sampling (Algorithm 1) is quadratic and depends on the iteration number k the number of data points (m and n), querying batch size b, and model architecture l.*

**Proof.**  Since Algorithm 1 is an iterative approach, the training time of the neural network is the main contributor to time complexity. On each iteration, the active learning agent has to perform the following steps:

1. Compute informativeness of the *m* unlabeled samples.
2. Cluster the *m* samples.
3. Find *b* samples with the highest informativeness.
4. Train the network with (n+kb) samples, where *k* is the iteration number.

The first two operations require O(m) time to complete, considering constant time for informativeness estimation. Clustering the *m* samples may vary in complexity depending on the clustering algorithm used. Assuming a standard clustering algorithm with a time complexity of O(m·c), where *c* is the complexity of the clustering algorithm, the total time complexity for step 2 is O(m·c).

If the training time increases linearly for (n+kb) samples, step 4 takes O((n+kb)l) time, where *l* is a term associated with the model architecture. So, the final time complexity for *k* iterations is:

(8) O(km+km·c+k(n+kb)l)→O(km+k(n+kb)l)

which is a quadratic complexity and comparable to that of a typical active learning framework. □

## 4. Experimental Setup

### 4.1. Datasets

To demonstrate our experiments, we chose KU-HAR [48] and DEAP [49] datasets.

KU-HAR contains smartphone recordings of 90 individuals’ (75 M, 15 F) 3-axial accelerometer and gyroscope data while performing 18 different activities. The samples were interpolated to maintain a 100 Hz sampling rate. To process the data, we adopted a sliding window of 5 s with 3 s overlap. The most frequent activity recognition window sizes are 0–1 s or 2–3 s [50]. The reasoning behind selecting a 5 s window is that lowering the window size further results in detecting atomic actions rather than activity behaviors. Furthermore, because some of the recordings are only 7 s long, adopting a larger window size would result in significant data loss.

Activity classification is a well-suited problem in mobile health. Therefore, to make the mentioned dataset compatible with multitask learning, we split it into three tasks: (1) classification by activity type, (2) classification by Metabolic Equivalent of Task (MET) values, and (3) classification by stress-inducing/relieving/neutral type (Table 1). MET values, as defined by the American Council on Exercise, measure how many calories are burned during any exercise. Hence, for task 2, we collected MET values [51] and divided the 18 activities into distinct subcategories [52]. Similarly, for task 3, all of these activities were categorized as stress-inducing, relieving, or highly relieving [53,54,55].

In DEAP, 32 participants (19–37 years, mean = 26.9, 50% females) watched 40 one-minute long music videos with different emotional orientations while their 32 channel electroencephalograph (EEG) and peripheral physiological signals (PPG, respiratory, GSR, temperature, etc.) were recorded at a rate of 128 Hz. Signals were filtered with a band-pass filter with the cut-off set at 4.0 and 45.0 Hz. DEAP includes user-defined ratings for valence, arousal, dominance, and liking, which we used as target emotions and their levels.

Each signal was segmented into 5 s long windows with an overlap of 2 s, and 70 features, explicitly mentioned in Table 2, were extracted from each segment using an EEG analysis tool [56,57]. Given that mood sensing is a well-matched task in mobile health setups [58], the function of the multitask model was organized to predict the high or low level of (1) valence, (2) arousal, and (3) dominance based on a threshold of 5 on all dimensions as three different tasks (Table 3).

### 4.2. Experiment

We chose a one-dimensional convolutional neural network (CNN)-based *hard-parameter sharing* multitask scheme [5] for KU-HAR. At the initial stages, when the labeled pool was tiny, a shallow model with one layer and eight filters was used to avoid overfitting the model as much as possible. As more labeled data became available, we gradually increased the number of neurons and layers in the predictive model across iterations to enhance its learning capacity. The selection of neurons and layers was guided by two criteria: ensuring convergence of training and test loss when sufficient labeled data were available or minimizing their difference as much as possible during initial iterations when labeled data were scarce. The final model had three layers, with each having 128 filters. The $l_{2}$ regularizer was placed with a factor of 0.01, while dropout layer rates ranged between 0.45 and 0.98 depending on the amount of data. Softmax activation was employed in output layers for multiclass classification under each task, and ReLU [59] was used in hidden layers. Class weights were either set manually or the training set was augmented to deal with unbalanced data. The number of epochs was consistent across all budget levels, i.e., 150 for KU-HAR.

For DEAP, our choice was a self-attention convolutional LSTM model of three layers with a *hard-parameter sharing* scheme of multitask network. Neuron numbers and dropout rate intensified as more data were introduced. Sigmoid activation was used in output layers under each task, while ReLU was employed in hidden layers. The $l_{2}$ regularization rate (0.01) and 800 epochs were consistent across all budgets.

Seed data consisted of three samples for initial training of the models. No preference was placed while selecting the seed samples, which means that every time, at least one class was missing a sample.

All the models were trained with an AMD Ryzen 7 2700X Eight-core CPU of 3.7 GHz speed, an NVIDIA GeForce GTX 1660 Ti Graphics Processing Unit (GPU) and 16 gigabytes of RAM. Hyper-parameters were tuned on a trial-and-error basis. All results were generated after identifying the best set of hyper-parameters. Results were evaluated with an accuracy-based performance metric and all the reported accuracy values on the KU-HAR dataset are an average of five attempts, with an average of three attempts for the DEAP dataset.

## 5. Results

We aim to demonstrate the querying techniques of MTAL in a mobile health setup constrained with low seed data and a low query budget and elevate the performance with CSS. This section highlights and analyzes the performances of all algorithms (one-sided, alternating, RC, CSS) at different query levels in terms of accuracy. Additionally, a comparison based on training time is conducted to examine if time is being compromised for better performance. We set several baselines in order to extensively validate the capability of CSS:

- ***One-sided selection*** selects samples or sensor observations with respect to only one learning task, assuming that the performance of other learners will also improve as we gather labels about those tasks. This approach is an intrinsic selection for the reference learner and an extrinsic selection for all others. We set the task with maximum combined correlation as the reference task:
  (9) $$t^{*}=\underset{t_{i}}{argmax}\;\sum_{j=1,j\neq i}^{T}corr\left(t_{i},t_{j}\right)$$
- ***Alternating selection*** alternates the reference task over the training process to identify critical samples. This approach is not biased towards a particular learner.
- ***Rank combination (RC)-based selection*** involves assigning *T* (= number of tasks) ranks, denoted as $r_{i}^{\left(k\right)}$, to each sample from the set $U^{m}$. The ranks are determined based on the entropy observed after the model makes predictions on these samples. Lower confidence margin or higher entropy means higher informativeness and, therefore, lower rank. Finally, the ranks belonging to each sample are summed up to develop a combined ranking:
  (10) $$R_{i}=\sum_{k=1}^{T}r_{i}^{\left(k\right)}$$
  The samples with the least combined rank are the top candidates for annotation.

### 5.1. Validity of Multitask Network Performance

From the basics of multitask models, deploying a hard-parameter sharing model requires the affiliated tasks to be closely related [12]. Since there is no concrete way to analyze task relationship [12,60], we first demonstrate that classification performance increased compared to that of single-task models as we incorporated multitask models. While supervised single-task accuracy numbers for KU-HAR were 93.4%, 92.6%, and 91.7% for classification by activity type, MET values, and stress, respectively, multitask registered 95.5%, 95.6%, and 93.6% under the same setup (dataset and task division), which were significant improvements from single-task. For DEAP, accuracy numbers were 70.4%, 70% and 70.5% in valence, arousal, and dominance level classification with single-task and slightly improved to 70.6%, 69.8%, and 71.2% after multitask deployment. These improvements validate the use of the multitask or hard-parameter sharing model for the aforementioned datasets and tasks.

Additionally, employing an STL for each task required higher combined execution time (6.9, 7.06, 6.9 min for task 1, 2, and 3 of KU-HAR, respectively, and 21.4, 21.5, 21.5 min for three tasks of DEAP). Also, STLs needed more parameters (291 k and 9 k for each task in two datasets, respectively) to train. In contrast, employing one MTL for all tasks ensured shorter execution time and training fewer parameters (9.9 min and 294 k parameters for KU-HAR and 23.8 min and 13.2 k for DEAP), which ensures a significant upgrade.

Furthermore, considering that the KU-HAR dataset encompasses 18 distinct labeled activities, one might contemplate an 18-class classification approach, subsequently translating it into multiple decisions arising from various tasks. However, this classification resulted in reduced accuracy, reaching only 74%.

### 5.2. Comparison Among One-Sided, Alternating and RC Sampling

We demonstrated one-sided sampling for different reference tasks and compared them against alternating and RC-based sampling at different querying budgets for both datasets. As mentioned earlier, the active learning agent iteratively utilizes the latest knowledge acquired by the multitask model to identify the best instances from the unlabeled pool.

**Figure 3:**
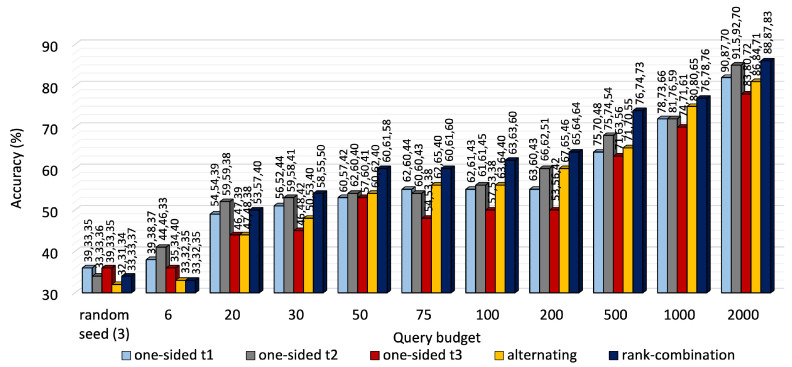
Accuracy comparison of one-sided, alternating and RC selection on KU-HAR at different querying budgets. The bars denote average of three task accuracy values placed above them.

**Figure 4:**
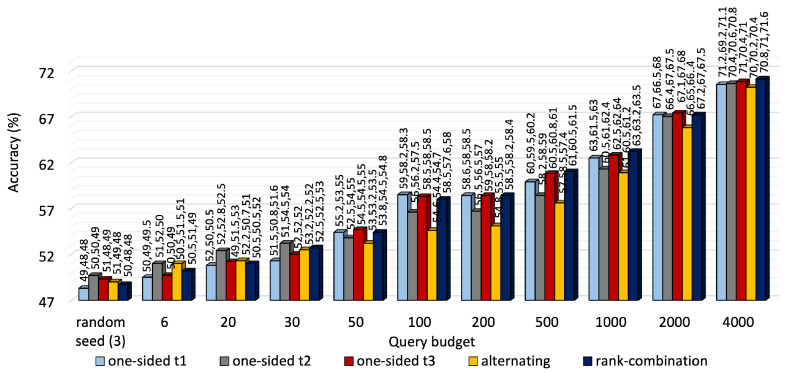
Accuracy comparison of one-sided, alternating and RC selection on DEAP at different querying budgets. The bars denote average of three task accuracy numbers placed above them.

A performance comparison of the three approaches is shown in Figure 3 for KU-HAR and Figure 4 for DEAP. For most budget levels, RC-based selection outperforms its counterparts. For example, at 2000 queries, i.e., almost one-fifth of the available unlabeled KU-HAR data, RC sampling achieved 88%, 87% and 83% accuracy levels for three tasks, surpassing alternating sampling—which obtained 86%, 84% and 71% accuracy—and one-sided sampling—which obtained the best record of 91.5%, 92% and 70%—under the same settings. Again, at 4000 queries, RC sampling reached 70.8%, 71% and 71.6% accuracy values, respectively, for three tasks in DEAP against 70%, 70.2% and 70.4% of alternating sampling and 71%, 70.4% and 71% of one-sided sampling (task 3 as reference). As expected, RC seldom prioritized a single task like one-sided sampling; rather, it tried to improve performance for all tasks regardless. While it is quite evident from Figure 3 that task 3 of KU-HAR achieves substantial improvements, it is capped at 72% for one-sided sampling with task 3 as reference. A similar improvement took place for task 2 of the DEAP dataset.

We notice that task 2 of KU-HAR, when set as the reference task of one-sided selection, performed the best among other reference tasks in terms of average accuracy (≈85%). Our observation is that task 2 for KU-HAR has the highest combined correlation with the other two tasks. Hence, labeling based on task 2 promoted shared learning and elevated the performance better than the other two. As shown in Table 4, the combined correlation of task 2 is 1.73, compared to 1.55 and 4.68 for task 1 and 2, respectively. For DEAP, task 3 shows the highest combined correlation (0.66) and the best results (average 70.8%) among other the reference task of one-sided selection.

### 5.3. Performance of CSS

**Figure 5:**
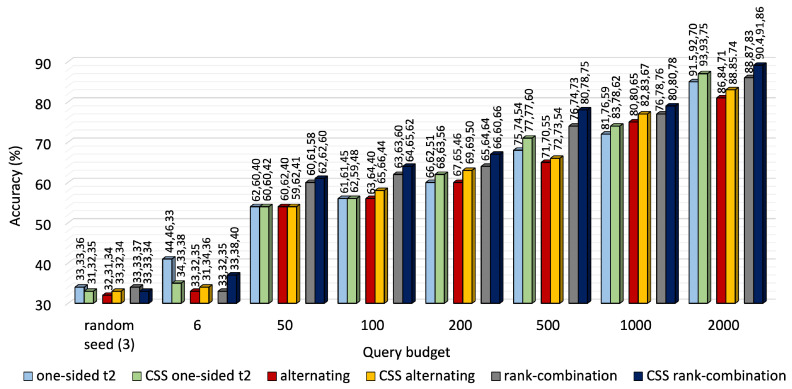
Performance comparison of one-sided, alternating and RC selection against their CSS versions for KU-HAR. One-sided selection is depicted with task 2 as reference since it showed best results among others. Average accuracy across 3 tasks improved at 2000 queries from 86% to 89.1% when CSS was launched with RC sampling. The bars indicate average of three task accuracy numbers above them.

**Figure 6:**
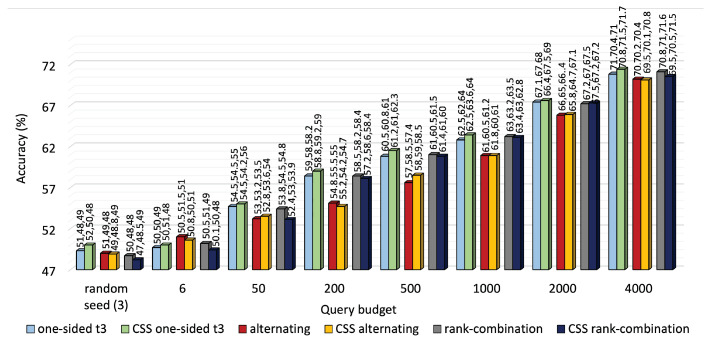
Performance comparison of CSS against its baseline counterparts for DEAP. Task 3 was set as reference task for one-sided selection as it produced best results among others. Average accuracy increases from 70.8% to 71.4% when CSS is attached to one-sided sampling and remains almost the same for other approaches. The bars depict average of three task accuracy values placed above them.

We implemented the clustered stratified sampling (CSS) algorithm for one-sided, alternating, RC sampling and compared their significance against those of plain one-sided, alternating and RC sampling at different budgets. Figure 5 and Figure 6 depict more analyses for KU-HAR and DEAP, respectively.

For KU-HAR, we formed three clusters on the unlabeled data. CSS outperformed the baseline selection schemes at most budget levels. At 2000 queries, CSS raised the average accuracy levels of one-sided, alternating and RC sampling to, respectively, 87%, 82.3%, 89.1% from 85%, 81%, 86%, achieved with their plain forms. Hence, CSS with RC sampling ensures a cumulative 9.3% accuracy gain over plain RC sampling.

We went up to 2000 prompts in the plots for KU-HAR, which may appear cumbersome to users. In mobile health, however, data will be generated from all 90 users of the KU-HAR dataset, yielding an average of 23 prompts per individual. Given a two-day data collection period, it would not be troublesome for users and would be compatible with µEMA.

While CSS improved the performance for one-sided selection on DEAP, such improvements were not visible for alternating and RC sampling. CSS depends on the performance of the clustering approach. Given the fact that the tasks of KU-HAR are highly correlated, clustering the unlabeled samples worked well for all tasks in all sampling approaches and helped CSS to identify the critical samples while maintaining equal distribution of samples from all classes. However, tasks of DEAP, not being highly correlated, could not make the most of clustering. Consequently, only one-sided sampling with task 3, with the DEAP dataset as reference, benefited from CSS (average accuracy jumped from 70.8% to 71.4%), and the other two sampling approaches suffered. Two clusters were formed with the unlabeled pool of DEAP, keeping it consistent with the number of classes.

### 5.4. Is Iterative Interaction Helping?

By the definition of active learning, there must be repetitive interaction between the active learning agent and the oracle. The agent will always train the multitask model with newly acquired labels and utilize the new knowledge to identify the most critical samples iteratively. However, iterative algorithms are computationally expensive. Thus, the question arises of whether repeated contact is truly worthwhile.

To address this, we created a lookalike version of MTAL that operates in a non-iterative mode, with only one interaction between the agent and the oracle regardless of budget. This way, the multitask model is trained with initial seed samples, the agent identifies the least confident samples from the unlabeled pool using the knowledge of the multitask model, contacts the oracle, labels the critical samples based on the querying budget, and finally trains the model.

**Figure 7:**
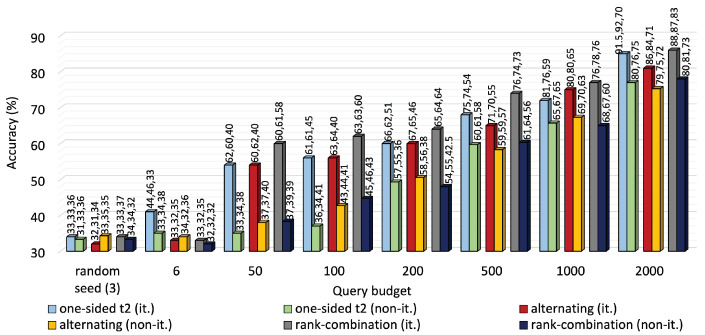
Average accuracy comparison between iterative and non-iterative versions of one-sided, alternating and RC sampling on KU-HAR dataset. Although iterative approaches are computationally expensive, they have outperformed the non-iterative approaches by substantially large margins.

**Figure 8:**
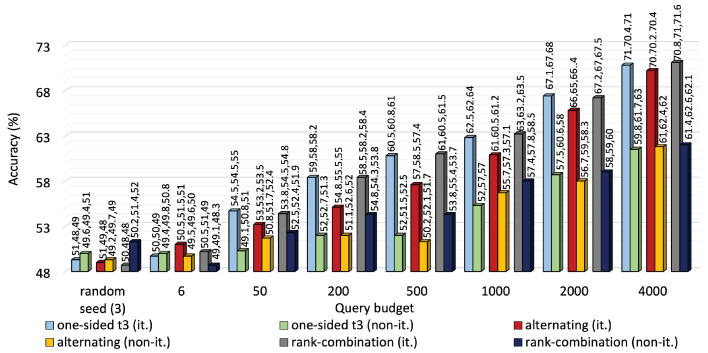
Average accuracy comparison between iterative and non-iterative versions of one-sided, alternating and RC sampling on DEAP dataset. Iterative approaches have outperformed the non-iterative approaches by large margins.

Figure 7 and Figure 8 compare the performance of iterative MTAL to its non-iterative counterpart on the two datasets. Average accuracy declined to 77%, 75.3% and 78% from 85%, 81% and 86%, respectively, for one-sided, alternating and RC sampling at 2000 queries on KU-HAR. Also, iterative leverage of new knowledge ensured over 10% higher average accuracy than non-iterative modes of all querying methods at the highest query level on DEAP.

The summary of performances on the two datasets is enlisted in Table 5 and Table 6, respectively.

Similarly, iterative exploitation of new knowledge guaranteed almost 10% higher average accuracy than non-iterative modes of all querying techniques at maximum query level on the DEAP dataset.

### 5.5. Training Time Comparison

**Figure 9:**
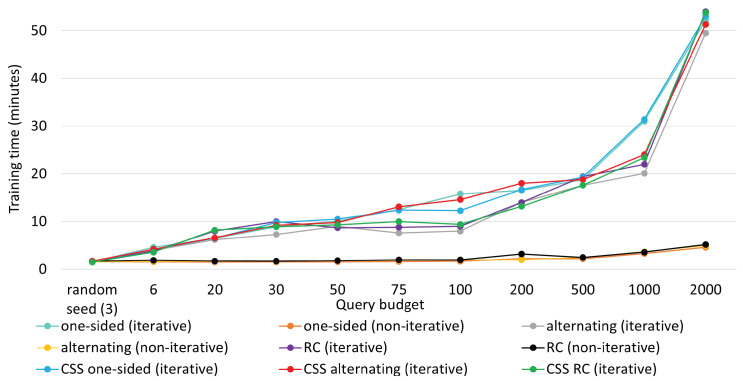
Training time comparison for all methods on KU-HAR. Training time also includes the time needed to find the best samples.

**Figure 10:**
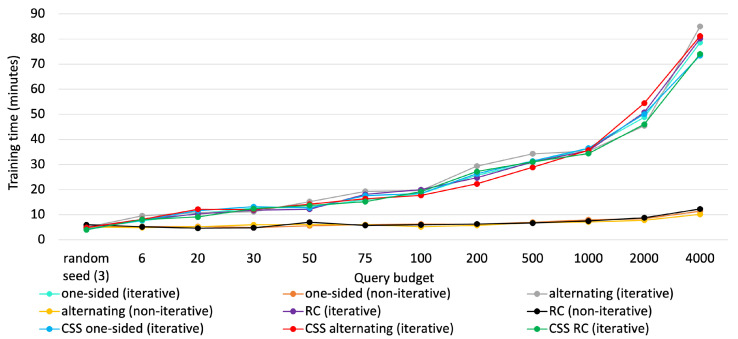
Training time comparison for all strategies on DEAP.

Lastly, we compared training times for the aforementioned methods in Figure 9 and Figure 10, respectively for the two datasets. Note that training time includes sampling time as well, be it an iterative process or a non-iterative one. Thereby, all the iterative mini-batch methods exhibited higher training time. In contrast, the non-iterative ones were trained faster, as new knowledge was not considered to sample critical instances and no repetitive interplay took place between the agent and the annotator.

For iterative queries, training time increased almost linearly until 1000 queries. As the model was made deeper to handle 2000 instances, training time jumped abruptly to 52 min, making it somewhat exponential. Training times remained constant for non-iterative methods. Leveraging the iterative process, MTAL obtained an edge over non-iterative methods at the expense of reasonably higher training time. The plots below also suggest that clustering in CSS does not compromise much in terms of training time.

## 6. Conclusions

We addressed the challenge of multitask active learning within mobile health contexts by introducing a novel framework called Clustered Stratified Sampling (CSS) for generating active learning queries. CSS can seamlessly integrate with any selection method and offer versatility in application. We emphasized CSS’s competitive performance against others. Importantly, we curated two benchmark datasets specifically tailored for multitask learning, focusing on activity and emotion analysis. An optimal reference task of one-sided sampling was discovered using statistical analysis. Additionally, we observed that inclusion of clustering into the CSS technique led to significant accuracy improvements without significantly extending training time. Our next objective is to further reduce the number of queries while maintaining performance and managing time complexity effectively.

## Figures and Tables

**Table 1:** Division of tasks for KU-HAR dataset.

Task 1—Classification by Activity Type		Task 2—Classification by MET Values		Task 3—Classification by Stress	
Stationary	stand, sit, talk-sit, talk-stand, lay	Light-intensity activities (MET < 3 )	stand, sit, talk-sit, talk-stand, lay	stress inducing	stand, sit, talk-sit, talk-stand (considering negative talk)
Activity of daily life (ADL)	stand-sit, lay-stand, stair-up, stair-down	Moderate-intensity activities (MET 3–6)	stand-sit, lay-stand, all walks, table tennis	stress relieving	stand-sit, lay, lay-stand, all walks
Exercise	pick, jump, push-up, sit-up, all walks, run, table tennis	Vigorous-intensity activities (MET ≥ 6)	pick, jump, push-up, sit-up, run, stair-up, stair-down	highly stress relieving	pick, jump, push-up, sit-up, run, stair-up, stair-down, table tennis

**Table 2:** Selected features from EEG signals of DEAP dataset.

Signal(s)	Corresponding Features
EEG	Average PSD in theta (4–8 Hz), low-alpha (8–10 Hz), alpha (8–12 Hz), high beta (12–30 Hz), and gamma (30–45 Hz) bands for 14 EEG channels: AF3, F3, F7, FC5, C3, P7, O1, AF4, F4, F8, T8, P8, O2 (5 powers × 14 channels)

**Table 3:** Task distribution for DEAP dataset.

Task 1—Valence Level Classification		Task 2—Arousal Level Classification		Task 3—Dominance Level Classification	
Low valence	valence < 5	Low arousal	arousal ≤ 5	low dominance	dominance < 5
High valence	valence ≥ 5	high arousal	arousal > 5	high dominance	dominance ≥ 5

**Table 4:** Correlation comparison and best reference task selection for one-sided selection. The highest combined correlation is highlighted in bold.

Correlations Among Different Tasks of KU-HAR				
	**Task 1**	**Task 2**	**Task 3**	**Combined Corr.**
task 1	-	0.8	0.75	1.55
task 2	0.8	-	0.927	**1.73**
task 3	0.75	0.927	-	1.68
**Correlations Among Different Tasks of DEAP**				
	**Task 1**	**Task 2**	**Task 3**	**Combined Corr.**
task 1	-	0.15	0.4	0.55
task 2	0.15	-	0.26	0.41
task 3	0.4	0.26	-	**0.66**

**Table 5:** Comparing the average accuracy (%) of different approaches at multiple query budgets on KU-HAR dataset. The highest accuracy values are highlighted in bold.

Query Budget	One-Sided	CSS One-Sided	Alternating %	CSS Alternating	Rank Combination	CSS Rank Combination
100	55.67	56.33	55.67	58.33	62	**63.67**
200	59.67	62.33	59.33	62.67	**64.33**	64
500	67.67	74.67	65.33	66.33	74.33	**77.67**
1000	72	54.33	75	77.33	76.67	**79.33**
2000	84.17	87	80.33	82.33	86	**89.13**

**Table 6:** Comparing the average accuracy (%) of different approaches at multiple query budgets on DEAP dataset. The highest accuracy values are highlighted in bold.

Query Budget	One-Sided	CSS One-Sided	Alternating %	CSS Alternating	Rank Combination	CSS Rank Combination
200	58.4	**59**	55.1	54.7	58.4	58.1
500	60.8	**61.5**	57.6	58.5	61	60.8
1000	**67.67**	63.4	60.9	60.1	63.2	63.1
2000	65.4	**67.63**	65.8	65.87	67.23	67.3
4000	70.87	**71.33**	70.2	70.13	71.13	70.5

## Data Availability

The datasets used in this article were derived from the following resources available in the public domain: KU-HAR: https://data.mendeley.com/datasets/45f952y38r/5 (accessed on 27 September 2024), DEAP: https://www.eecs.qmul.ac.uk/mmv/datasets/deap/index.html (accessed on 27 September 2024).
